# Evaluation of resistance mechanisms and susceptibility testing in *Enterococcus* spp.: implications of vancomycin-variable isolates and *optrA*-mediated resistance

**DOI:** 10.1007/s10096-026-05556-8

**Published:** 2026-06-02

**Authors:** Mervenur Demir, Aylin İrem Ocaklı, Elif Seren Tanrıverdi, Gülşen Hazırolan, Alpaslan Alp, Barış Otlu, Burçin Şener

**Affiliations:** 1 Department of Medical Microbiology, Inonu University, Malatya, Türkiye; 2 Department of Medical Microbiology, University of Health Science Etlik City Hospital, Ankara, Türkiye; 3 Department of Medical Microbiology, Hacettepe University, Ankara, Türkiye

**Keywords:** *Enterococcus*, VRE, OptrA, Vancomycin, Linezolid, Antibiotic resistance

## Abstract

**Purpose:**

Vancomycin-resistant enterococci (VRE) represent a major challenge in healthcare settings due to limited therapeutic options and the emergence of resistance to last-line agents. Accurate detection of resistance mechanisms and understanding the molecular epidemiology of circulating strains are essential for effective surveillance and infection control.

**Methods:**

A total of 192 non-duplicate *Enterococcus* isolates (103 VRE and 89 vancomycin-susceptible isolates) recovered from clinical specimens between 2018 and 2022 were analyzed. Antimicrobial susceptibility testing was performed using broth microdilution (BMD) as the reference method and compared with automated and gradient-based methods. Resistance genes associated with vancomycin and linezolid resistance were investigated by in-house PCR and sequencing. Clonal relatedness of VRE isolates was assessed using AP-PCR, PFGE, and MLST.

**Results:**

Among VRE isolates, *vanA* was the predominant resistance determinant, while a single isolate carried *vanB*. Importantly, *vanA* was also detected in three phenotypically vancomycin-susceptible *E. faecalis* isolates, consistent with vancomycin-variable enterococci. Linezolid resistance was identified in three isolates (1.6%); two *E. faecalis* isolates harbored the transferable *optrA* gene, representing the first report of *optrA*-positive *E. faecalis* causing human infections in Türkiye, while one *E. faecium* isolate showed linezolid resistance in the absence of the investigated resistance genes. Tigecycline resistance was uncommon. Daptomycin retained in vitro activity with higher MIC₅₀/ MIC₉₀ values in *E. faecium*, whereas oritavancin MIC values were higher among *E. faecalis* isolates. The BD Phoenix system demonstrated high categorical agreement with BMD for vancomycin and teicoplanin but generated major and very major errors for linezolid susceptibility testing. Molecular typing revealed extensive genetic diversity among VRE isolates, with 35 AP-PCR genotypes, PFGE-confirmed clustering including one predominant clone, and the identification of 11 novel MLST sequence types (ST2994–ST3004).

**Conclusions:**

This study provides insight into the resistance landscape and genetic diversity of clinical *Enterococcus* isolates. The detection of vancomycin-variable enterococci and transferable linezolid resistance determinants underscores the need for integrated phenotypic and molecular approaches to ensure accurate resistance detection and effective surveillance.

**Supplementary Information:**

The online version contains supplementary material available at https://doi.org/10.1007/s10096-026-05556-8.

## Introduction

*Enterococcus faecalis* and *Enterococcus faecium* are among the leading causes of healthcare-associated infections, including endocarditis, septicemia, urinary tract infections, and wound infections [1]. These organisms exhibit intrinsic resistance to several antimicrobial classes, such as cephalosporins, sulfonamides, macrolides and low levels of aminoglycosides [2]. Beyond their intrinsic resistance and tolerance, enterococci readily acquire resistance to most antimicrobials used in clinical practice. This is largely due to their genomic plasticity, which enables the acquisition of resistance determinants that enhance colonization and pathogenicity [1]. The extensive use of vancomycin and other broad-spectrum antibiotics has driven the emergence of vancomycin-resistant enterococci (VRE), most notably in *E. faecium* [3]. Vancomycin-resistant *E. faecium* (VREfm) is among the high-priority pathogens in the 2024 World Health Organization (WHO) Bacterial Priority Pathogens List, highlighting the limited availability of effective therapeutic options [4]. Healthcare-associated *E. faecium* isolates are typically resistant to ampicillin, and the prevalence of vancomycin resistance varies geographically. The 2023 European Centre for Disease Prevention and Control (ECDC) Annual Epidemiological Report indicates that the population-weighted prevalence of VREfm in the EU/EEA increased from 16.2% in 2020 to 19.8% in 2023, with national rates ranging between 0.0% and 60.9% [5]. According to the latest report on antimicrobial resistance surveillance in Europe by the ECDC and the WHO, based on 2021 data, the prevalence of VREfm in Türkiye was reported as 15.8% [6].

*Van* gene clusters represent the most common mechanism of acquired vancomycin resistance. To date, nine distinct types of acquired glycopeptide resistance (*vanA*,* vanB*,* vanD*,* vanE*,* vanG*,* vanL*,* vanM*,* vanN*, and *vanP*) have been described in enterococci, with the *vanA* and *vanB* genotypes being the most prevalent. The *vanA* gene cluster confers high-level, inducible resistance to both vancomycin and teicoplanin, whereas *vanB* confers variable levels of resistance to vancomycin only, leaving susceptibility to teicoplanin unaffected, with resistance inducible exclusively by vancomycin [7]. More recently, vancomycin-variable enterococci (VVE)—which harbor the *vanA* gene but display phenotypic susceptibility—have been described and are particularly concerning due to their potential to revert to high-level resistance during therapy [8]. The detection and clinical interpretation of these isolates remain challenging in routine laboratories, necessitating molecular testing alongside phenotypic susceptibility assays.

Linezolid, an oxazolidinone antibiotic, is considered one of the last therapeutic options for treating VRE and other multidrug-resistant gram-positive infections. However, resistance to linezolid has been increasingly reported over the past decade. Two mechanisms mainly mediate linezolid resistance in enterococci. Linezolid resistance is mainly mediated by mutations in the 23 S rRNA gene and by alterations in ribosomal proteins L3/L4, which affect the oxazolidinone-binding site [9, 10]. Additionally, plasmids and chromosomally integrated mobile genetic elements carrying the *cfr*,* optrA*, or *poxtA* genes contribute to the dissemination of oxazolidinone resistance [11, 12]. Daptomycin remains a key agent for VRE, but challenges with susceptibility testing and rising resistance restrict its use. While newer agents such as oritavancin and tigecycline show in vitro activity, clinical evidence and standardized testing data are still limited [3]. The concurrent emergence of resistance to multiple last-line agents such as linezolid, daptomycin, and tigecycline in *Enterococcus* spp. severely limits therapeutic options and underscores the critical need for accurate resistance detection. This study aimed to characterize the antimicrobial susceptibility profiles and resistance genotypes of vancomycin-resistant and -susceptible *Enterococcus* isolates from clinical samples, to assess the performance of various antimicrobial susceptibility testing (AST) methods compared to the reference broth microdilution (BMD) method, and to explore the genetic relatedness of resistant isolates through molecular typing.

## Materials and methods

### Bacterial isolates

The enterococci isolated from the clinical specimens at the Central Bacteriology Laboratory of Hacettepe University Hospitals, Ankara, Türkiye, between January 2018 and December 2022 were included in this study. Among these, 103 vancomycin-resistant *Enterococcus* spp. (VRE) and 89 randomly selected vancomycin-susceptible *Enterococcus* spp. (VSE) isolates were examined. To avoid duplication, only one isolate per patient was analyzed. All isolates were identified to species level with a score of ≥ 2.0 by MALDI-TOF-MS Biotyper 4.1 (Bruker Daltonics, GmbH, Bremen, Germany).

### Antimicrobial susceptibility testing

Ampicillin, ciprofloxacin, high-level streptomycin resistance (HLSR), high-level gentamicin resistance (HLGR), vancomycin, teicoplanin, and linezolid susceptibility results of the isolates determined by the BD Phoenix (Becton Dickinson, USA) automated AST system were obtained from the Laboratory Information Management System data.

Vancomycin, teicoplanin, linezolid, daptomycin, tigecycline, and oritavancin susceptibility profiles of the isolates were detected by the BMD utilizing cation-adjusted Mueller-Hinton broth (CAMHB) according to the ISO 20776-1 standard [13]. Mueller-Hinton broth with 50 µg/ml of calcium was used to determine daptomycin susceptibility, while Mueller-Hinton broth supplemented with 0.002% polysorbate-80 was used to assess oritavancin susceptibility. The CAMHB used for tigecycline BMD was prepared fresh on the day of use. *Staphylococcus aureus* ATCC 29,213 and *Enterococcus faecalis* ATCC 29,212 strains were used for quality control. To ensure comparability across methods, all MIC results (including those obtained from the BD Phoenix system) were interpreted according to EUCAST clinical breakpoints (v15.0, 2025) [14]. CLSI criteria were applied for daptomycin and oritavancin since there are no breakpoints in EUCAST (M100-ED35:2025) [15]. HLGR and HLSR were also categorized following CLSI criteria, as the BD-Phoenix cut-off values for these two antibiotics were based on CLSI guidelines. ECOFF values, which distinguish wild-type isolates from isolates with acquired resistance mechanisms, were used separately to describe MIC distributions and were not interpreted as clinical susceptibility breakpoints. MIC_50_ and MIC_90_ were defined as the minimum inhibitory concentrations inhibiting 50% and 90% of isolates, respectively.The results obtained from the BD Phoenix automated system were compared with the results of BMD applied as the reference method for vancomycin, teicoplanin and linezolid susceptibility testing. Gradient strip tests (ETest, bioMérieux, France) were performed for the isolates identified as resistant by the automated AST system (*n* = 103 for vancomycin, *n* = 103 for teicoplanin, and 6 for linezolid). Additionally, a 5 µg/mL vancomycin disc diffusion test was conducted for all isolates, and the results were compared with the BMD results.

The performance of different test methods was evaluated based on categorical agreement (CA), defined as the percentage of results within the same susceptibility category as the reference method. A very major error (VME) was defined as a false susceptible result (reporting S instead of R). A major error (ME) was defined as a false resistant result (reporting R instead of S). The VME and ME rates were calculated by dividing the number of errors by the total number of resistant and susceptible isolates, respectively, as determined by the reference method. Percentages ≥ 90% for CA, ≤ 3% for ME, and ≤ 3% for VME were considered acceptable [16].

### Molecular detection of resistance genes

Genomic DNA of the isolates was extracted by the boiling method [17]. An aliquot of the supernatant DNA (2 µL) was used as the template in a final volume of 20 µL polymerase chain reaction (PCR) mixture which contained: 13 µL water, 250 nM of each primer and 4 µL FIREPol Master Mix (Solis Biodyne, Tartu, Estonia). The presence of genes encoding vancomycin and linezolid resistance was determined by an in-house PCR method using previously described primers [18–21]. Table S1 lists the primers and amplification conditions. The amplified products were separated by gel electrophoresis using a 1.5% (w/v) agarose gel and visualized with the BioSpectrum 500 Imaging System (Upland, CA, USA) under ultraviolet light. The *vanA* and *vanB* genes were investigated in all isolates, while the *cfr*,* optrA*, and *poxtA* genes were examined in linezolid-resistant *Enterococcus* spp. (LRE) isolates. The isolates in which the linezolid resistance gene was detected were confirmed by sequencing. During the PCR product purification step, single-band samples were purified using the MAGBIO HighPrep™ PCR Clean-up System (MagBio Genomics Europe, Kraichtal, Germany) according to the manufacturer’s instructions. For Sanger sequencing, an ABI 3730XL DNA Analyzer (Applied Biosystems, Foster City, CA, USA) and the BigDye Terminator v3.1 Cycle Sequencing Kit (Applied Biosystems) were employed.

Clonal relatedness of the VREfm isolates was investigated at the Molecular Microbiology Laboratory of Inonu University, Malatya, Türkiye. Arbitrarily primed polymerase chain reaction (AP-PCR) was performed for all VRE and LRE isolates. AP-PCR was performed as previously described by Kuzucu et al. [22]. Pulsed-field gel electrophoresis (PFGE) was subsequently applied to isolates sharing similar AP-PCR genotypes to confirm clonal relatedness, according to the protocol optimized by Turabelidze et al. [23]. Band profiles were analyzed with the GelCompar II software system (version 6.6; Applied Maths, Sint-Martens-Latem, Belgium) (optimization 1.0, tolerance 1.0, cut off %90). The similarity for band analysis was calculated using the Dice correlation coefficient and UPGMA (Unweighted Pair Group Method with Arithmetic Mean) was used for cluster analysis. Based on the combined AP-PCR and PFGE findings, representative isolates (*n* = 18) from predominant and distinct clonal groups were selected for multilocus sequence typing (MLST) analysis [24]. The sequences were analyzed using the PubMLST database to determine allele numbers and sequence types [25].

### Statistical evaluation

SPSS Software version 26 was employed for statistical analyses. Categorical variables, including resistance rates between groups, were compared using the chi-squared test or Fisher’s exact test when appropriate. Continuous variables, including MIC distributions, were analyzed using the Mann–Whitney U test. A significance threshold of 0.05 was established to assess statistical significance.

## Results

The study included a total of 192 isolates, which were obtained from various clinical samples: blood (*n* = 88), urine (*n* = 55), pus (*n* = 17), tissue/biopsy (*n* = 14), pleural fluid (*n* = 9), peritoneal fluid (*n* = 5), pericardial fluid (*n* = 1), bronchoalveolar lavage (*n* = 1), joint fluid (*n* = 1) and bile (*n* = 1). MALDI-TOF MS identified 145 isolates as *E. faecium* and 47 as *E. faecalis*. The species distribution differed markedly between the VRE and VSE groups: all VRE isolates were *E. faecium* (103/103), whereas the VSE group comprised 42 *E. faecium* and 47 *E. faecalis* isolates.

All VRE isolates exhibited resistance to teicoplanin and harbored the *vanA* gene, except for a single isolate that was teicoplanin-susceptible and carried the *vanB* gene. Additionally, all vancomycin-susceptible isolates were also susceptible to teicoplanin. The *vanA* gene was detected in 3 out of 89 (3.3%) vancomycin-susceptible isolates. Notably, all VVE isolates were identified as *E. faecalis*.

**Fig. 1:**
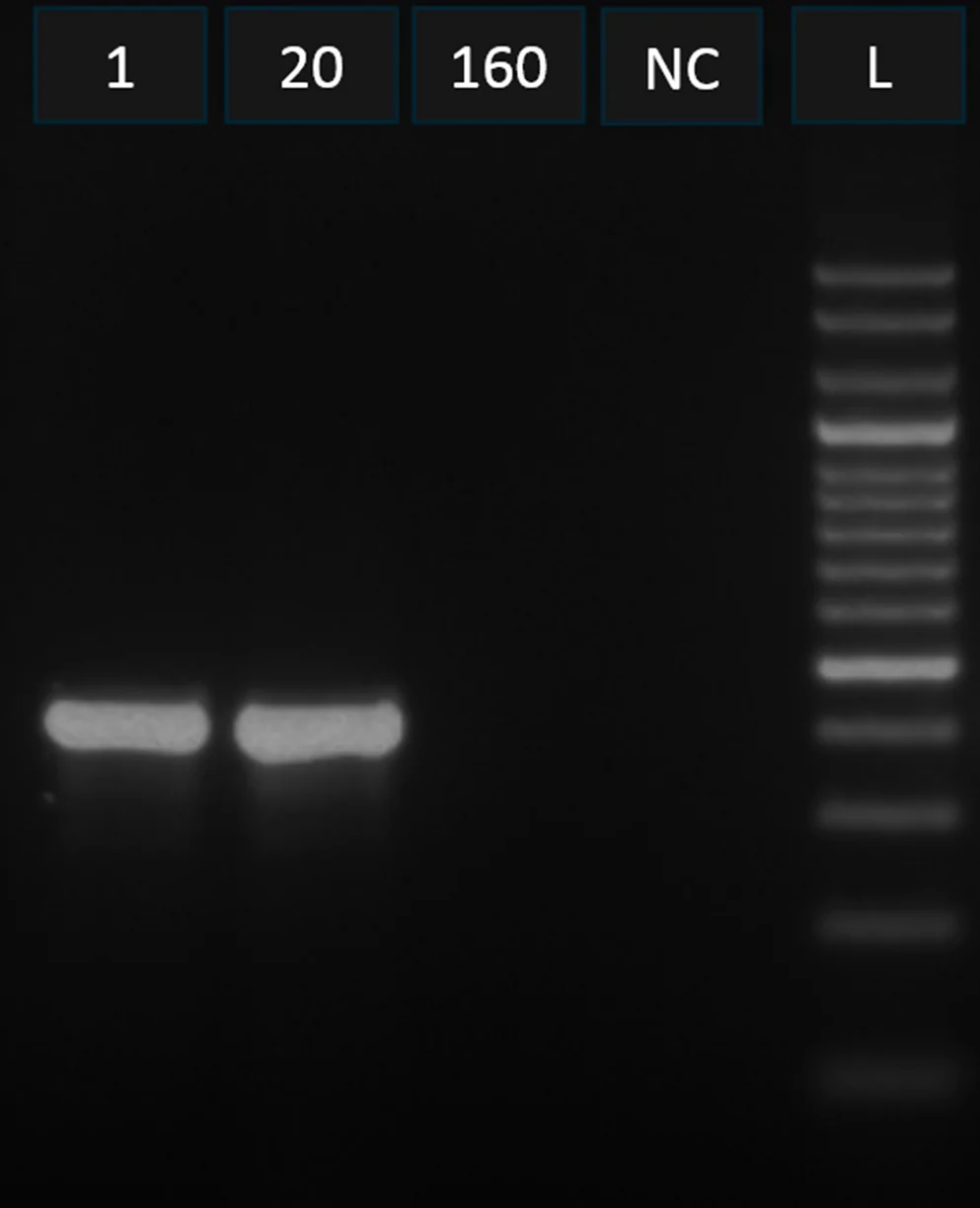
The agarose gel electrophoresis (1.5%) image shows the *optrA* PCR products from linezolid-resistant isolates 1, 20, and 160. The expected product size is 422 bp. Isolates 1 and 20 tested positive for the *optrA* gene, while isolate 160 was negative. The lanes labeled “NC” and “L” correspond to negative (blank) control and the ladder, respectively. The ladder used is the 100 bp DNA Ladder Ready to Load (Solis Biodyne, Tartu, Estonia)

Linezolid resistance was detected in two *E. faecalis* (1.1%) and one *E. faecium* (0.5%) isolate based on BMD results (Table 1). Linezolid-resistant *E. faecium* isolate was also resistant to vancomycin, teicoplanin, daptomycin and tigecycline. The *optrA* gene was found in two linezolid-resistant *E. faecalis* isolates using PCR (Fig. 1) and was confirmed by sequence analysis (percent identity: 100%, GenBank accession number: KX620937.1). The *cfr* and *poxtA* genes were negative in all LRE isolates.

The resistance rates for ampicillin (100% vs. 38.2%), ciprofloxacin (86.4% vs. 41.6), HLGR (62.1% vs. 46.1%) and HLSR (77.7 vs. 52.8) were significantly higher in VRE isolates compared to vancomycin-susceptible *Enterococcus* (VSE) isolates (*p* < 0.05) (Table S2). Tigecycline resistance was identified in four (2.1%) *E. faecium* isolates, three of which were also vancomycin resistant. The differences in tigecycline and linezolid resistance rates between *E. faecium* and *E. faecalis* isolates were not statistically significant (*p* > 0.05) (Table 1). Moreover, no statistically significant difference in tigecycline and linezolid resistance rates was observed between VRE and VSE isolates (*p* > 0.05). There are no breakpoints in the EUCAST Clinical Breakpoint Tables v. 15.0 for daptomycin and oritavancin. All *Enterococcus* spp. isolates showed MIC values below the EUCAST ECOFF value for daptomycin (8 mg/L for *E. faecium* and 4 mg/L for *E. faecalis*). The *E. faecium* isolates exhibited higher MIC_50_ and MIC_90_ values for daptomycin compared to the *E. faecalis* isolates (*p* < 0.001). Seven *E. faecalis* isolates had MIC values exceeding the ECOFF value (0.06 mg/L) for oritavancin. Currently, no ECOFF value has been established for *E. faecium* concerning oritavancin. The *E. faecalis* isolates had higher MIC_50_ and MIC_90_ values for oritavancin than the *E. faecium* isolates (*p* < 0.001). Four *E. faecium* isolates (3.9%) were resistant to daptomycin, while four *E. faecalis* isolates (8.5%) were classified as intermediate based on CLSI breakpoints. Two *E. faecalis* isolates and one *E. faecium* isolate exhibited MIC values above CLSI interpretive thresholds for oritavancin. The MIC_50_ and MIC_90_ values, MIC ranges, and resistance rates are presented in Table 1.

**Table 1:** Antibiotic susceptibility testing results of E. faecium and E. faecalis isolates

Species	Antibiotic	Test Range (mg/L)	MIC Range (mg/L)	MIC_50_ (mg/L)	MIC_90_ (mg/L)	R, *n* (%)
*Enterococcus faecium* (*n* = 145)	Ampicillin	2–16	≤ 2->16	> 16	> 16	137 (94.5)
	Ciprofloxacin	1–4	≤ 1->4	> 4	> 4	113 (77.9)
	HLGR	500	≤ 500->500	-	-	92 (63.4)*
	HLSR	1000	≤ 1000->1000	-	-	108 (73.8) *
	Vancomycin	0.25–128	≤ 0.25->128	> 128	> 128	103 (71.0)
	Teicoplanin	0.125-64	≤ 0.125->64	32	> 64	102 (70.3)
	Linezolid	0.25–128	≤ 0.25-32	1	2	1 (0.7)
	Tigecycline	0.016-8	≤ 0.016-4	0.06	0.125	4 (2.8)
	Daptomycin	0.06-32	0.125-8	2	4	4 (2.8) *
	Oritavancin	0.004-2	≤ 0.004–0.25	≤ 0.004	0.016	1 (0.6) *^NS^
*Enterococcus faecalis* (*n* = 47)	Ampicillin	2–16	≤ 2	≤ 2	≤ 2	0 (0)
	Ciprofloxacin	1–4	≤ 1->4	≤ 1	> 4	13 (27.6)
	HLGR	500	≤ 500->500	-	-	13 (27.6)*
	HLSR	1000	≤ 1000->1000	-	-	19 (40.4) *
	Vancomycin	0.25–128	≤ 0.25-4	0.5	2	0 (0)
	Teicoplanin	0.125-64	≤ 0.125-1	0.25	0.5	0 (0)
	Linezolid	0.25–128	≤ 0.25-8	1	2	2 (4.3)
	Tigecycline	0.016-8	≤ 0.016–0.25	0.06	0.125	0 (0)
	Daptomycin	0.06-32	0.125-4	1	2	0 (0)*
	Oritavancin	0.004-2	≤ 0.004–0.25	0.016	0.125	2 (4.2)*^NS^

*HLGR*, High-level gentamicin resistance; *HLSR*, high-level streptomycin resistance; *NS*, nonsusceptible

*CLSI breakpoints were applied

Resistance profiles were also examined according to species and vancomycin susceptibility status (Table S3). VREfm isolates showed significantly higher resistance rates to ampicillin (100% vs. 80.9%, *p* < 0.01) and ciprofloxacin (86.4% vs. 57.1%, *p* < 0.01) compared with vancomycin-susceptible *E. faecium* isolates, whereas HLGR and HLSR rates were similar between the two groups (*p* > 0.05). In addition, vancomycin-susceptible *E. faecalis* isolates demonstrated significantly lower resistance rates to ampicillin, ciprofloxacin, HLGR, and HLSR compared with vancomycin-susceptible *E. faecium* isolates (*p* < 0.05 for all).

This study evaluated the performance of the BD Phoenix automated system and the disk diffusion method in determining antibiotic susceptibility of *Enterococcus* spp., using the reference BMD method for comparison. The frequency of ME observed for vancomycin with BD Phoenix system was 1.1% and VME was 1.9%, whereas no VME or ME was detected with the gradient test or disk diffusion method (Table 2). The CA rates of the BD Phoenix system, gradient test, and disk diffusion method with BMD were 98.4%, 100%, and 100%, respectively. The isolate with a ME was not a VVE (van PCR-negative), showing a vancomycin MIC of 2 mg/L by BMD and 16 mg/L by BD Phoenix. When compared with BMD, BD Phoenix system yielded no ME or VME for teicoplanin susceptibility, while gradient test revealed one ME (1.1%) and one VME (0.9%). The CA rates of the BD Phoenix system and the gradient test with BMD were 100% and 98.1%, respectively. In terms of linezolid susceptibility testing, four MEs (2.1%) and one VME (33.3%) were generated by the BD Phoenix system, whereas the gradient test revealed only one VME (33.3%), with no observed MEs. The CA rates of the BD Phoenix system and gradient test with BMD were 97.4% and 83.3%, respectively.

**Table 2:** Comparison of different antibiotic susceptibility testing methods in *Enterococcus* spp. isolates with the reference broth microdilution method

Antibiotic	Method	Categorical agreement (%)	ME, *n* (%)	VME, *n *(%)
Vancomycin	Disk diffusion	100	0	0
	BD Phoenix	98.4	1 (1.1)	2 (1.9)
	Gradient test^a^	100	0	0
Teicoplanin	BD Phoenix	100	0	0
	Gradient test^a^	98.1	1 (1.1)	1 (0.9)
Linezolid	BD Phoenix	97.4	4 (2.1)	1 (33.3)
	Gradient test^b^	83.3	0 (0)	1 (33.3)

n, number; %, percent

^a^The gradient tests were applied to 103 isolates identified as resistant to teicoplanin and/or vancomycin by BD Phoenix

^b^The gradient tests were applied to six isolates identified as resistant by BD Phoenix

The dendrogram created by analyzing the band profiles of AP-PCR using the GelCompar II software system is shown in Figure S1. AP-PCR revealed 35 distinct genotypes for VRE and three distinct genotypes for LRE isolates. VRE isolates revealed 16 diverse clusters (tolerance 1.0, optimization 1.0, cutoff: 90%). Of all the isolates, 84 were located in any cluster, and the clustering rate was 81.5%. The largest cluster consisted of genotype 12, which contained 23 isolates. The clustering patterns obtained by AP-PCR were further supported by PFGE analysis. Based on the combined AP-PCR and PFGE results, isolates showing distinct PFGE banding patterns together with representatives of predominant AP-PCR clusters were selected for MLST analysis. Accordingly, a total of 18 representative isolates were included in the MLST scheme to reflect the overall genetic diversity of the collection while avoiding repetitive analysis of clonally indistinguishable isolates. As presented in Table S4, the selected isolates included representatives from major clusters as well as unique or less frequent genotypes. The MLST scheme identified ST1661 in one isolate. A total of 11 novel sequence types identified in this study were deposited in the PubMLST database, where new ST numbers (ST2994–ST3004) were assigned accordingly [25]. Several sequence types were represented by multiple isolates, including ST2996 (*n* = 23), ST3002 (*n* = 11), and ST2997 (*n* = 9). In addition, several sequence types were represented by a single isolate. ST2997, ST2998, ST3000, and ST3002 were identified in isolates belonging to more than one AP-PCR genotype.

### Discussion

In this study, we analysed the antimicrobial resistance profiles, resistance determinants, and clonal relationships of *E. faecalis* and *E. faecium* isolates, with a particular focus on vancomycin and linezolid resistance. Consistent with previous studies, all vancomycin-resistant isolates in our cohort, except one, harbored the *vanA* gene and were co-resistant to teicoplanin, while the single *vanB*-positive isolate remained susceptible to teicoplanin. This resistance pattern is characteristic of *vanA*-mediated resistance, which typically confers high-level resistance to both vancomycin and teicoplanin, whereas *vanB* is often associated with variable resistance to vancomycin and preserved susceptibility to teicoplanin [26–28]. Of particular interest was the identification of three *E. faecalis* isolates carrying *vanA* that remained phenotypically susceptible to vancomycin, commonly referred to as VVE. Such isolates may be missed by routine phenotypic susceptibility testing [7, 8]. Supporting this concern, Coccitto et al. demonstrated that *vanA*-positive VVE isolates may harbor alterations within the regulatory regions of the Tn1546 transposon, resulting in reduced gene expression despite retention of the resistance cluster and enabling phenotypic reversion under glycopeptide exposure [29]. The presence of VVE in our cohort highlights the limitations of relying solely on phenotypic testing and underscores the need for molecular screening strategies in surveillance and outbreak settings to prevent the silent dissemination of glycopeptide resistance within healthcare environments.

Linezolid resistance was detected in only three isolates (1.6%), representing a relatively low rate consistent with previous reports from Europe and Türkiye [30, 31]. Earlier national data from Türkiye documented linezolid resistance in 1.8% of enterococcal isolates, indicating that resistance may be encountered at low rates in clinical settings [32]. A recent systematic review by Wada et al. reported pooled prevalence rates of linezolid-resistant *Enterococcus* (LRE) of 1.9% (95% CI: 1.3–2.8%) in humans and 6.3% (95% CI: 3.1–12.3%) in animals. The predominant resistance mechanism reported was *optrA*, followed by *cfr* and *poxtA* [31]. In our study, molecular analysis identified the *optrA* gene in two *E. faecalis* isolates, a transferable resistance determinant conferring resistance to both linezolid and phenicols. To the best of our knowledge, this represents the first report of *optrA*-positive *E. faecalis* causing human infections in Türkiye. This finding aligns with recent studies that suggest the increasing global dissemination of *optrA*, particularly among *E. faecalis* strains [12, 33]. Notably, one *E. faecium* isolate showed linezolid resistance without harboring any of the resistance genes tested. The absence of the investigated resistance genes indicates that other resistance mechanisms may be involved; however, mutations in the 23 S rRNA or ribosomal proteins L3/L4 were not investigated in the present study [3, 31]. Therefore, the underlying mechanism of linezolid resistance in the gene-negative isolate could not be fully elucidated. Our findings support the need for continued surveillance of LRE isolates to monitor the spread of these mobile resistance elements.

In the present study, tigecycline resistance was observed at a very low rate in *E. faecium* isolates (2.8%), while no resistant *E. faecalis* isolates were detected, consistent with international surveillance reports where resistance among enterococci generally remains below 5% [33, 34]. Recent data from Türkiye similarly reported tigecycline resistance rates of 1.2% in *E. faecium* and 0.4% in *E. faecalis*, with no linezolid resistance detected [35]. Tigecycline resistance in enterococci is typically linked to mutations in the ribosomal protection protein Tet(M) or upregulation of efflux pumps, underscoring the potential for adaptive resistance under selective pressure [33]. These findings support the continued in vitro activity of tigecycline against enterococci, although ongoing surveillance remains important.

The evaluation of alternative therapeutic options, such as daptomycin and oritavancin, is essential given the limited choices for multidrug-resistant enterococci. While daptomycin retained potent in vitro activity against both species, in our study, *E. faecium* showed significantly higher MIC₅₀ and MIC₉₀ values than *E. faecalis*. Daptomycin resistance was detected in 2.8% (*n* = 4) of *E. faecium* isolates; notably, all four resistant isolates were *VRE.* International surveillance studies demonstrate that more than 99.6% of enterococcal isolates remain susceptible to daptomycin worldwide [33, 34]. However, emerging evidence indicates rising rates of daptomycin nonsusceptibility in VRE, particularly in institutions with extensive drug use, with prevalence reported up to 21% [36, 37]. Similar to global trends, our finding of resistant *E. faecium* and intermediate *E. faecalis* underscores the importance of optimized dosing and combination strategies to sustain daptomycin effectiveness against VRE.

Interpretation of oritavancin susceptibility in *Enterococcus* spp. remains challenging. Under the CLSI framework, testing and reporting are restricted to vancomycin-susceptible *E. faecalis*, limiting applicability to *E. faecium* and VRE isolates. EUCAST has not established clinical breakpoints for oritavancin, and ECOFF values are available only for *E. faecalis* (0.06 mg/L). In our study, *E. faecalis* isolates showed higher MIC values than *E. faecium*, with seven exceeding the ECOFF. Two *E. faecalis* and one *E. faecium* isolate had MIC values above CLSI thresholds, suggesting that reduced susceptibility, although rare, is not negligible. However, given these limitations, categorical interpretation should be approached with caution. In a study of 1,086 enterococcal isolates, vancomycin-intermediate strains had very low oritavancin MICs (0.004–0.015 mg/L), and VRE isolates displayed MICs of 0.002–1 mg/L [38]. Likewise, the recent SENTRY surveillance program documented oritavancin susceptibility rates of 92.2–98.3% among VRE worldwide [34]. To date, Turkish data on oritavancin are scarce, with only one in vitro study from İstanbul University evaluating VREfm isolates and reporting low MICs (0.015–0.24 mg/L) [39]. Taken together, our findings highlight species-specific differences and the variability in MIC distributions, emphasizing the need for harmonized breakpoints and further clinical data to clarify the role of oritavancin in enterococcal infections.

Additional analysis according to species and vancomycin susceptibility status showed that higher ampicillin and ciprofloxacin resistance rates persisted among VREfm isolates compared with vancomycin-susceptible *E. faecium* isolates, whereas HLGR and HLSR rates were similar between the two groups. In contrast, vancomycin-susceptible *E. faecalis* isolates generally exhibited lower resistance rates, consistent with known species-related susceptibility patterns. However, comparisons between VRE and VSE isolates should be interpreted cautiously, given the marked difference in species distribution between the groups.

Regarding the performance of susceptibility testing methods, the BD Phoenix system demonstrated high categorical agreement with the reference BMD method for vancomycin (98.4%) and teicoplanin (100%) susceptibility testing, aligning with prior evaluations of its reliability in detecting glycopeptide resistance [40, 41]. Commercial systems, such as BD Phoenix and Vitek 2, generally perform well in glycopeptide susceptibility testing of enterococci. However, while BD Phoenix shows consistently high accuracy, Vitek2 demonstrates lower reliability, particularly for *vanB*-positive isolates and strains with low-level, inducible resistance [3, 42]. Our findings show that the automated system detected vancomycin resistance in a *vanB*-positive isolate, yet the insufficient number of such isolates precludes a conclusive assessment of its diagnostic performance. These findings highlight the need for confirmatory testing and optimized screening approaches to avoid misdiagnosis and VRE transmission.

With respect to linezolid, although the BD Phoenix system showed high categorical agreement, reliable assessment of AST performance, particularly VME rates, was not possible because of the very limited number of resistant isolates. Nevertheless, the observed VME finding should not be overlooked [40, 41]. A study revealed that the automated methods failed to detect several resistant isolates carrying *optrA* and *poxtA*, thereby highlighting the risk of relying solely on automated systems for the detection of linezolid resistance [43]. In our study, although only two *optrA*-positive isolates were available, both were correctly identified as resistant by the BD Phoenix system. The gradient strip test showed perfect agreement for vancomycin (100%) and teicoplanin (98.1%), whereas agreement for linezolid was lower (83.3%); however, this finding should be interpreted with caution, as it was based on only six isolates.

Molecular typing demonstrated substantial genetic diversity among VRE isolates, with 35 AP-PCR genotypes and PFGE-confirmed clustering patterns. The distribution of isolates into multiple clusters indicates the coexistence of several circulating clones within the healthcare setting, including one predominant cluster representing approximately one-quarter of the isolates. While certain sequence types, such as ST2996 and ST3002, were represented by a large number of isolates, multiple singleton sequence types were also identified, reflecting a heterogeneous population structure. In addition, the detection of ST2998 across different AP-PCR genotypes highlights the presence of genomic variability within a single MLST sequence type. MLST was performed on representative isolates selected according to combined AP-PCR and PFGE findings. This strategy enabled characterization of the circulating sequence types in a cost-effective and epidemiologically meaningful manner while still capturing the major genetic diversity of the collection. The identification of several novel sequence types further highlights the local genetic diversity of VRE isolates and contributes additional data to global MLST databases. However, because MLST analysis was restricted to representative isolates rather than the entire collection, full sequence type resolution across all isolates could not be achieved, which should be considered when interpreting the molecular epidemiology findings.

This study has some limitations. It was conducted at a single center, which may limit the generalizability of the findings. The low number of isolates resistant to last-line agents such as linezolid, daptomycin, and oritavancin restricts detailed interpretation of resistance mechanisms and susceptibility testing performance. In addition, the unequal species distribution between the VRE and VSE groups represents a potential limitation for direct comparison of resistance profiles. A further limitation is that molecular analysis was limited to selected resistance genes, and mutations in the 23 S rRNA or ribosomal proteins were not investigated. Furthermore, detailed genomic characterization of daptomycin and tigecycline-nonsusceptible isolates and VVE was not performed. Therefore, the genetic basis of these phenotypes and the underlying resistance mechanisms could not be fully elucidated. Regarding MLST analysis, only representative isolates were included, which may have limited the resolution of clonal relationships despite providing an overview of circulating sequence types.

Overall, an integrated evaluation of antimicrobial resistance, resistance mechanisms, and molecular epidemiology provides insight into clinical *Enterococcus* isolates in a tertiary care setting in Türkiye. Vancomycin resistance was predominantly mediated by the *vanA* genotype, and the identification of vancomycin-variable enterococci highlights the limitations of phenotypic testing alone and the need for molecular confirmation. Although resistance to linezolid and tigecycline remained low, the detection of transferable resistance determinants such as *optrA* indicates the potential for further dissemination of resistance to last-line agents. Antimicrobial susceptibility testing methods showed generally high agreement with the reference broth microdilution method, while molecular typing revealed substantial genetic diversity. Together, these findings support the use of combined phenotypic and molecular approaches for accurate resistance detection, effective infection control, and ongoing surveillance of enterococcal infections.

## Supplementary Information

Below is the link to the electronic supplementary material.

Supplementary Material 1

## Data Availability

The datasets generated during the current study are available from the corresponding author on reasonable request.

## Abbreviations

AP-PCR: Arbitrarily primed polymerase chain reaction
AST: Antimicrobial susceptibility testing
BMD: Broth microdilution
CA: Categorical agreement
CAMHB: Cation–adjusted Mueller–Hinton broth
CLSI: Clinical and Laboratory Standards Institute
ECDC: European Centre for Disease Prevention and Control
ECOFF: Epidemiological cut–off value
EUCAST: European Committee on Antimicrobial Susceptibility Testing
HLGR: High–level gentamicin resistance
HLSR: High–level streptomycin resistance
LRE: Linezolid–resistant enterococci
MALDI-TOF MS: Matrix–assisted laser desorption ionization–time of flight mass spectrometry
ME: Major error
MIC: Minimum inhibitory concentration
MIC_50_: Minimum inhibitory concentration inhibiting 50% of isolates
MIC_90_: Minimum inhibitory concentration inhibiting 90% of isolates
MLST: Multilocus sequence typing
PCR: Polymerase chain reaction
PFGE: Pulsed–field gel electrophoresis
VME: Very major error
VRE: Vancomycin–resistant enterococci
VREfm: Vancomycin–resistant Enterococcus faecium
VSE: Vancomycin–susceptible enterococci
VVE: Vancomycin–variable enterococci
WHO: World Health Organization
