# Supplementary material for: Evaluation of resistance mechanisms and susceptibility testing in Enterococcus spp.: implications of vancomycin-variable isolates and optrA-mediated resistance
Source: Eur J Clin Microbiol Infect Dis. 2026 Jun 2;45(9):2791–800. doi: 10.1007/s10096-026-05556-8 (PMC13593646; doi:10.1007/s10096-026-05556-8)
Supplement: Supplementary file 1 [file 10096_2026_5556_MOESM1_ESM.docx]

Table S1. Sequences of primers used for PCR amplification of the *vanA, vanB, cfr, optrA* and *poxtA* genes

| Gene | Primer | Sequence (5ʹ→3ʹ) | Product size (bp) | Reference | PCR conditions |
| --- | --- | --- | --- | --- | --- |
| *vanA* | vanA-F | GGGAAAACGACAATTGC | 732 | 1 | (95 °C, 5 min) x 1 cycle; (95 °C, 30 s; 56 °C, 60 s; 72 °C, 60 s) x 35 cycles; (72 °C, 10 min) x 1 cycle |
|  | vanA-R | GTACAATGCGGCCGTTA |  |  |  |
| *vanB* | vanB-F | ATGGGAAGCCGATAGTC | 635 | 1 | (95 °C, 5 min) x 1 cycle; (94 °C, 30 s; 55 °C, 30 s; 72 °C, 30 s) x 35 cycles; (72 °C, 5 min) x 1 cycle |
|  | vanB-R | GATTTCGTTCCTCGACC |  |  |  |
| *cfr* | cfr-F | TGAAGTATAAAGCAGGTTGGGAGTCA | 746 | 2 | (95 °C, 5 min) x 1 cycle; (95 °C, 30 s; 50 °C, 30 s; 72 °C, 30 s) x 35 cycles; (72 °C, 5 min) x 1 cycle |
|  | cfr-R | ACCATATAATTGACCACAAGCAGC |  |  |  |
| *optrA* | optrA-F | TACTTGATGAACCTACTAACCA | 422 | 3 | (95 °C, 5 min) x 1 cycle; (95 °C, 30 s; 50 °C, 30 s; 72 °C, 30 s) x 35 cycles; (72 °C, 5 min) x 1 cycle |
|  | optrA-R | CCTTGAACTACTGATTCTCGG |  |  |  |
| *poxtA* | poxtA-F | GAACGCTTGGAGTATTTCGACTTC | 778 | 4 | (95 °C, 5 min) x 1 cycle; (95 °C, 30 s; 50 °C, 30 s; 72 °C, 30 s) x 35 cycles; (72 °C, 5 min) x 1 cycle |
|  | poxtA-R | CTGGACTGAGAATACCCATC |  |  |  |

Table S2. Comparison of antibiotic susceptibility results of VRE and VSE isolates

| Species | Antibiotic | Test Range (mg/L) | MIC Range (mg/L) | MIC_50_  (mg/L) | MIC_90_  (mg/L) | R, n(%) |
| --- | --- | --- | --- | --- | --- | --- |
| VRE (n=103) | Ampicillin | 2-16 | 16->16 | >16 | >16 | 103 (100) |
|  | Ciprofloxacin | 1-4 | ≤1->4 | >4 | >4 | 89 (86.4) |
|  | HLGR | 500 | ≤500->500 | - | - | 64 (62.1)* |
|  | HLSR | 1000 | ≤1000->1000 | - | - | 80 (77.7)* |
|  | Linezolid | 0.25-128 | ≤0.25-32 | 1 | 1 | 1 (0.9) |
|  | Tigecycline | 0.016-8 | ≤0.016-4 | 0.06 | 0.125 | 3 (2.9) |
|  | Daptomycin | 0.06-32 | 0.125-8 | 2 | 4 | 4 (3.9)* |
|  | Oritavancin | 0.004-2 | ≤0.004-0.25 | ≤0.004 | 0.016 | 1 (0.9)*^NS^ |
| VSE  (n=89) | Ampicillin | 2-16 | ≤2->16 | ≤2 | >16 | 34 (38.2) |
|  | Ciprofloxacin | 1-4 | ≤1->4 | 2 | >4 | 37 (41.6) |
|  | HLGR | 500 | ≤500->500 | - | - | 41 (46.1)* |
|  | HLSR | 1000 | ≤1000->1000 | - | - | 47 (52.8)* |
|  | Linezolid | 0.25-128 | ≤0.25-8 | 1 | 2 | 2 (2.2) |
|  | Tigecycline | 0.016-8 | ≤0.016-4 | 0.06 | 0.125 | 1 (1.1) |
|  | Daptomycin | 0.06-32 | 0.125-4 | 1 | 4 | 0 (0)* |
|  | Oritavancin | 0.004-2 | ≤0.004-0.25 | 0.008 | 0.06 | 2 (2.2)*^NS^ |
| VRE, Vancomycin-Resistant Enterococcus; Vancomycin-Susceptible Enterococcus; HLGR, High-level gentamicin resistance; HLSR, high-level streptomycin resistance; NS, nonsusceptible.  *CLSI breakpoints were applied. | | | | | | |

Table S3. Species-stratified antimicrobial susceptibility profiles of vancomycin-resistant and vancomycin-susceptible Enterococcus isolates

| Species | Antibiotic | Test Range (mg/L) | MIC Range (mg/L) | MIC_50_  (mg/L) | MIC_90_  (mg/L) | R, n(%) |
| --- | --- | --- | --- | --- | --- | --- |
| Vancomycin resistant *E. faecium* (n=103) | Ampicillin | 2-16 | 16->16 | >16 | >16 | 103 (100) |
|  | Ciprofloxacin | 1-4 | ≤1->4 | >4 | >4 | 89 (86.4) |
|  | HLGR | 500 | ≤500->500 | - | - | 64 (62.1)* |
|  | HLSR | 1000 | ≤1000->1000 | - | - | 80 (77.7)* |
|  | Linezolid | 0.25-128 | ≤0.25-32 | 1 | 1 | 1 (0.9) |
|  | Tigecycline | 0.016-8 | ≤0.016-4 | 0.06 | 0.125 | 3 (2.9) |
|  | Daptomycin | 0.06-32 | 0.125-8 | 2 | 4 | 4 (3.9)* |
|  | Oritavancin | 0.004-2 | ≤0.004-0.25 | ≤0.004 | 0.016 | 1 (0.9)*^NS^ |
| Vancomycin susceptible *E. faecium*  (n=42) | Ampicillin | 2-16 | ≤2->16 | >16 | >16 | 34 (80.9) |
|  | Ciprofloxacin | 1-4 | ≤1->4 | >4 | >4 | 24 (57.1) |
|  | HLGR | 500 | ≤500->500 | - | - | 28 (66.7)* |
|  | HLSR | 1000 | ≤1000->1000 | - | - | 28 (66.7)* |
|  | Linezolid | 0.25-128 | ≤0.25-2 | 1 | 2 | 0 (0) |
|  | Tigecycline | 0.016-8 | ≤0.016-4 | 0.03 | 0.125 | 1 (2.4) |
|  | Daptomycin | 0.06-32 | 0.25-4 | 2 | 4 | 0 (0)* |
|  | Oritavancin | 0.004-2 | ≤0.004-0.03 | ≤0.004 | 0.008 | 0 (0)* |
| Vancomycin susceptible *E. faecalis* (n=47) | Ampicillin | 2-16 | ≤2** | ≤2 | ≤2 | 0 (0) |
|  | Ciprofloxacin | 1-4 | ≤1->4 | ≤1 | >4 | 13 (27.6) |
|  | HLGR | 500 | ≤500->500 | - | - | 13 (27.6) |
|  | HLSR | 1000 | ≤1000->1000 | - | - | 19 (40.4) |
|  | Linezolid | 0.25-128 | ≤0.25-8 | 1 | 2 | 2 (4.2) |
|  | Tigecycline | 0.016-8 | ≤0.016-0.25 | 0.06 | 0.125 | 0 (0) |
|  | Daptomycin | 0.06-32 | 0.125-4 | 1 | 2 | 0 (0)* |
|  | Oritavancin | 0.004-2 | ≤0.004-0.25 | 0.015 | 0.125 | 2 (4.2)*^NS^ |
| HLGR, High-level gentamicin resistance; HLSR, high-level streptomycin resistance; NS, nonsusceptible.  *CLSI breakpoints were applied.  **All isolates’ MICs were below 2 mg/L | | | | | | |

Table S4. Relationship between AP-PCR genotypes and MLST sequence types with corresponding isolate counts

| Isolate number | AP-PCR genotype | Sequence type | Number of isolates with the same AP-PCR genotype |
| --- | --- | --- | --- |
| 113 | 13 | ST1661 | 6 |
| 6 | 26 | ST2994 | 6 |
| 111 | 17 | ST2995 | 1 |
| 117 | 12 | ST2996 | 23 |
| 130 | 21 | ST2997 | 1 |
| 153 | 1 | ST2997 | 8 |
| 131 | 23 | ST2998 | 1 |
| 145 | 11 | ST2998 | 2 |
| 163 | 2 | ST2998 | 8 |
| 186 | 9 | ST2998 | 2 |
| 148 | 22 | ST2999 | 1 |
| 151 | 19 | ST3000 | 1 |
| 159 | 29 | ST3000 | 1 |
| 164 | 20 | ST3001 | 2 |
| 168 | 30 | ST3002 | 1 |
| 173 | 5 | ST3002 | 10 |
| 169 | 15 | ST3003 | 1 |
| 183 | 35 | ST3004 | 1 |

**Figure S1.** The dendrogram obtained from AP-PCR analysis of VRE and LRE (1,20, 160) isolates. Genotypes are color-coded.

**Figure S1 (continued).** The dendrogram obtained from AP-PCR analysis of VRE and LRE (1,20, 160) isolates. Genotypes are color-coded.

**Figure S2.** AP-PCR gel image of isolates numbered 2, 4, 6, 7, 10, 11, 12, 18, 19, 21, 23, 27, 36, 37, 39, 41, and 42. DNA ladders were included as molecular size markers.

**Figure S3.** AP-PCR gel image showing isolates numbered 45, 101, 102, 103, 104, 105, 106, 107, 108, 109, 110, 111, 112, 113, 114, 115, and 116. DNA ladders were included as molecular size markers.

**Figure S4.** AP-PCR gel image showing isolates numbered 117, 118, 119, 120, 121, 122, 123, 124, 125, 126, 127, 128, 129, 130, 131, 132, 133. DNA ladders were included as molecular size markers.

**Figure S5.** AP-PCR gel image showing isolates numbered 134, 135, 136, 137, 138, 139, 140, 141, 142, 144, 145, 146, 148, 149, 150, 151, 152. DNA ladders were included as molecular size markers.

**Figure S6.** AP-PCR gel image showing isolates numbered 153, 154, 155, 156, 157, 158, 159, 160, 161, 162, 163, 164, 165, 166, 167, 168, 169. DNA ladders were included as molecular size markers.

**Figure S7.** AP-PCR gel image showing isolates numbered 173, 174, 175, 177, 178, 180, 181, 183, 185, 186, 187, 188, 189, 190, 192, 193, 194, 195. DNA ladders were included as molecular size markers.

**Figure S8.** AP-PCR gel image showing isolates numbered 170 and 172. DNA ladder was included as a molecular size marker.

**Figure S9.** PFGE Gel Image of Isolates 153 (M), 154, 156, 2, 7, 4, 12, M, 128, 129, 117, 139, 178, 180, M

**Figure S10**. PFGE Gel Image of Isolates M, 173, 175, 181, 185, 187, 103, M, 107, 105, 136, 137, 10, 23, M

**Figure S11.** PFGE Gel Image of Isolates M, 27, 102, 110, 112, 115, 123, M, 127, 140, 118, 120, 121, 116, M

**References:**

1. Dutka-Malen, S., Evers, S., & Courvalin, P. (1995). Detection of glycopeptide resistance genotypes and identification to the species level of clinically relevant enterococci by PCR. Journal of clinical microbiology, 33(1), 24–27.

2. Kehrenberg C, Schwarz S. Distribution of florfenicol resistance genes fexA and cfr among chloramphenicol-resistant Staphylococcus isolates. J Antimicrob Chemother 2006; 50: 1156-63.

3. Brenciani A, Morroni, Pollini S et al. Characterization of novel conjugative multiresistance plasmids carrying cfr from linezolid-resistant Staphylococcus epidermidis clinical isolates from Italy. J Antimicrob Chemother 2016; 71: 307–13.

4. Brenciani A, Fioriti S, Morroni G, et al. Detection in Italy of a porcine Enterococcus faecium isolate carrying the novel phenicol-oxazolidinone-tetracycline resistance gene poxtA. J Antimicrob Chemother. 2019;74(3):817-818.
